# Supplementary material for: Precision of a Hand-Held 3D Surface Scanner in Dry and Wet Skeletal Surfaces: An Ex Vivo Study
Source: Diagnostics (Basel). 2022 Sep 18;12(9):2251. doi: 10.3390/diagnostics12092251 (PMC9497896; doi:10.3390/diagnostics12092251)
Supplement: Supplementary file 1 [file diagnostics-12-02251-s001.zip › diagnostics-1824120-supplementary.pdf]

Precision of a hand-held 3D surface scanner in dry and wet skeletal surfaces: an ex vivo study

Jannis Probst, Konstantinos Dritsas, Demetrios Halazonetis, Yijin Ren, Christos Katsaros, Nikolaos Gkantidis

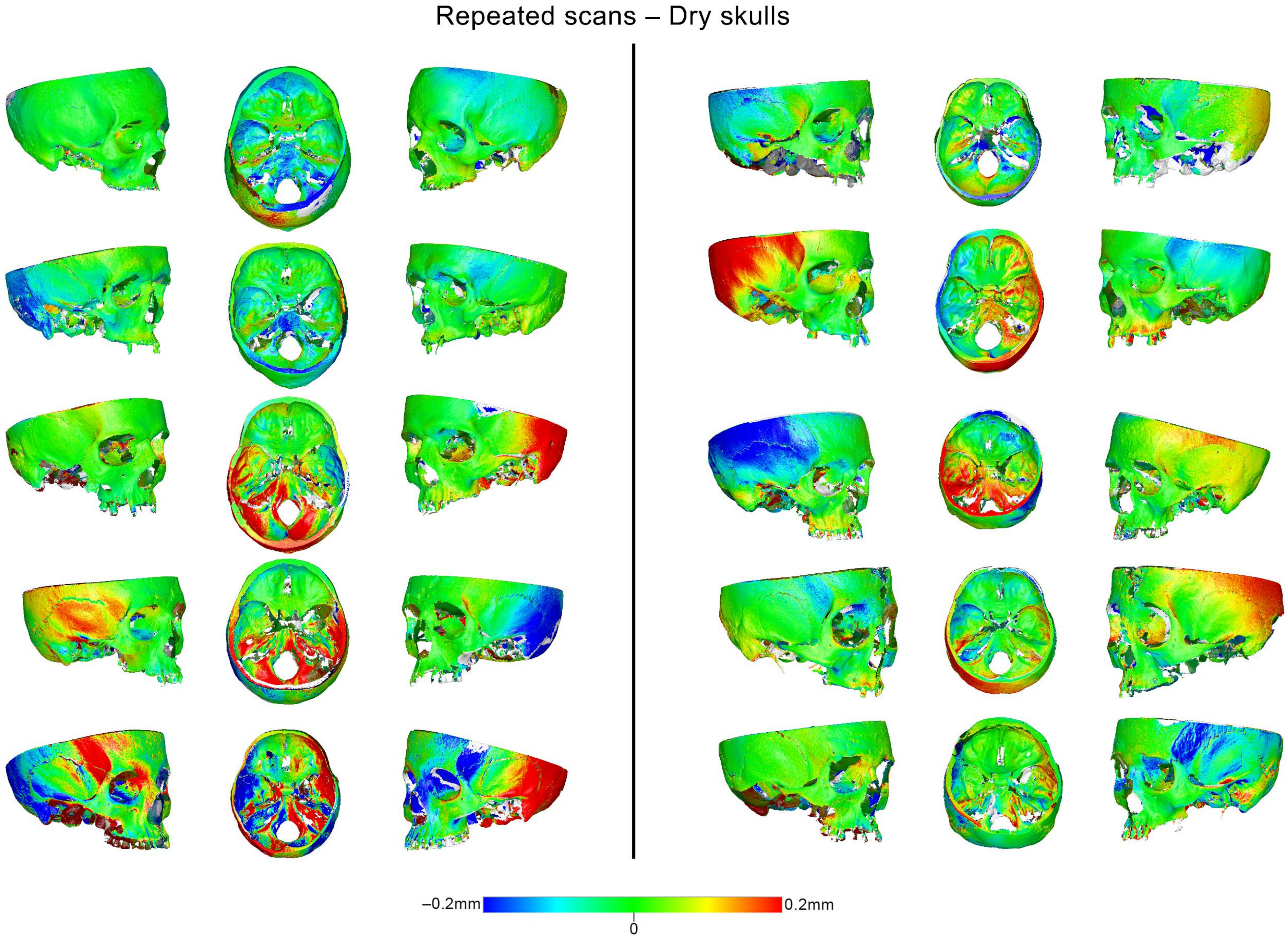

Figure S1. Color coded distance maps of best-fit superimpositions between repeated scans of 10 dry skulls. Three skull views in a row per side correspond to a single specimen.

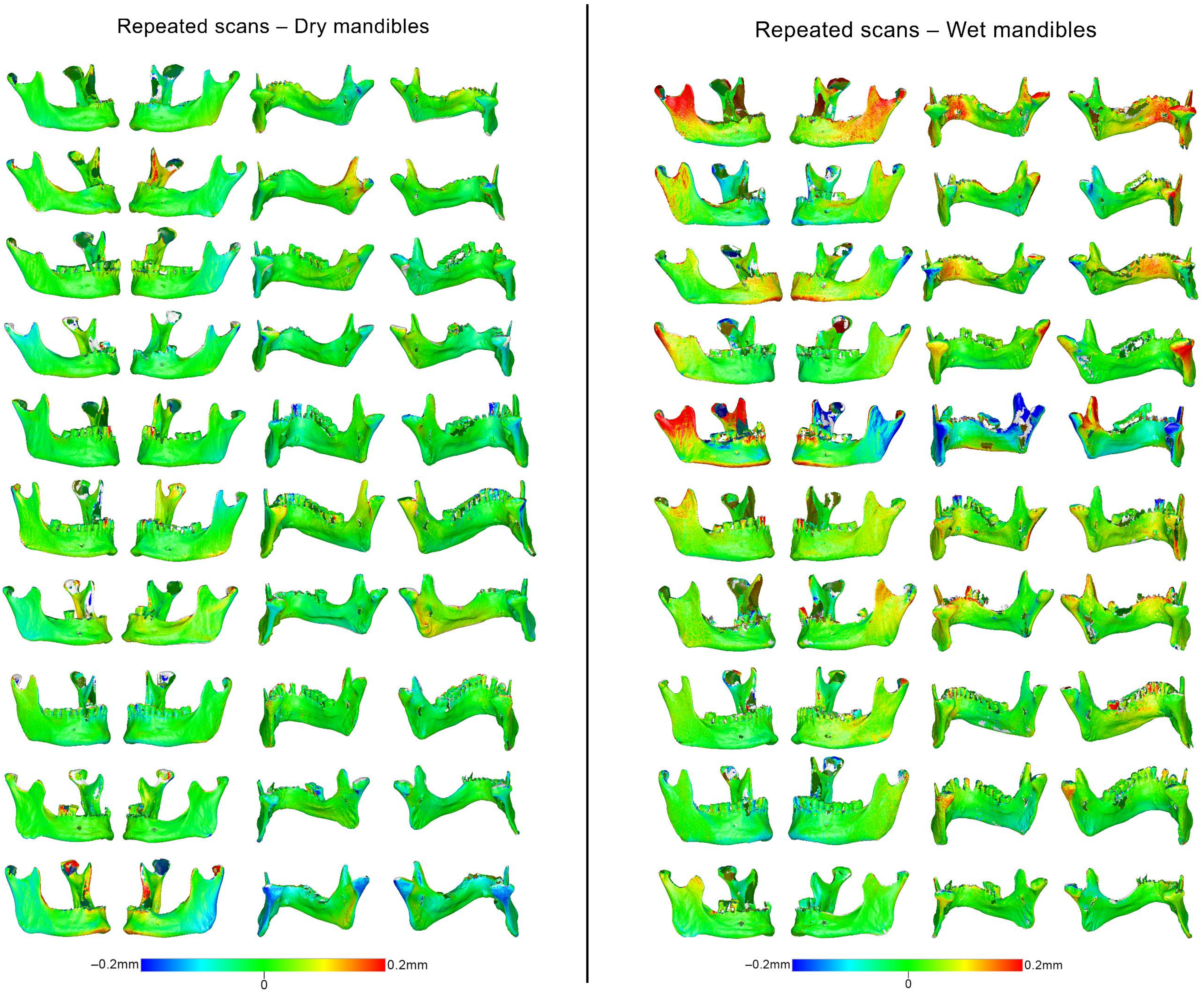

Figure S2. Color coded distance maps of best-fit superimpositions between repeated scans of 10 dry and 10 wet mandibles. Four mandibular views in a row per side correspond to a single specimen.

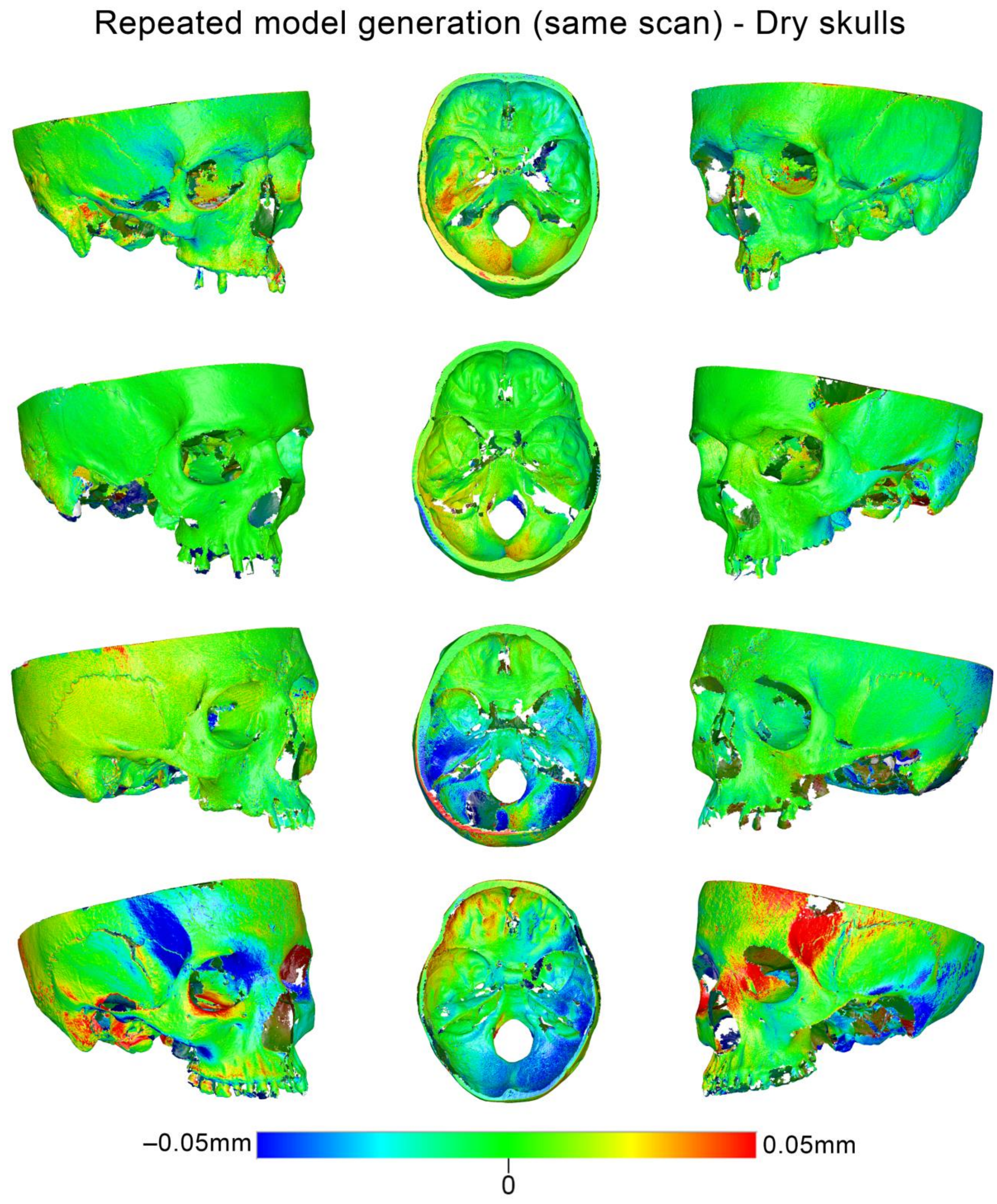

Figure S3. Color coded distance maps of best-fit superimpositions of repeated model generations of the same scan for 4 dry skulls. Three skull views in a row per side correspond to a single specimen.

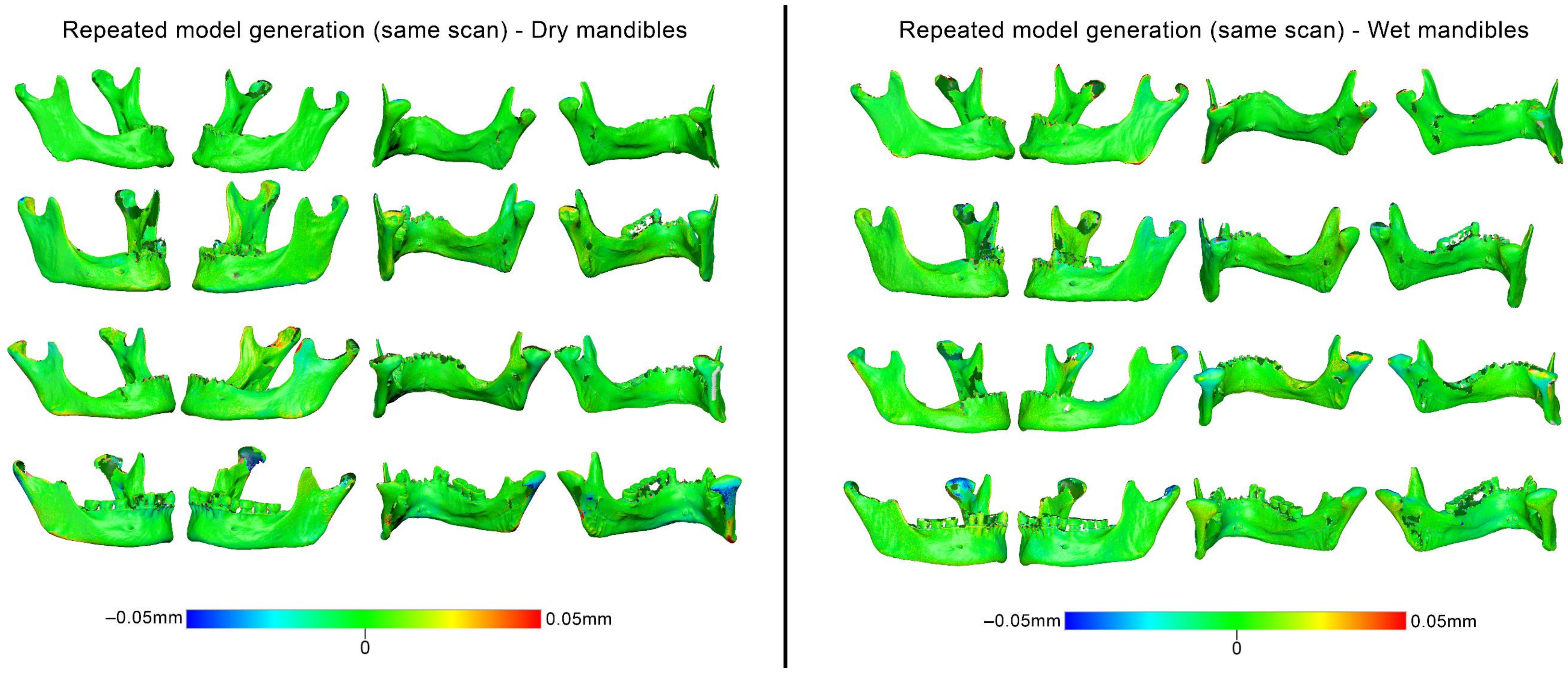

Figure S4. Color coded distance maps of best-fit superimpositions of repeated model generations of the same scan for 4 dry and 4 wet mandibles. Four mandibular views in a row per side correspond to a single specimen.
